# Diagnostic criteria and scoring systems for thyroid storm: An evaluation of their utility – comparative review

**DOI:** 10.1097/MD.0000000000037396

**Published:** 2024-03-29

**Authors:** Chukwuka Elendu, Dependable C. Amaechi, Emmanuel C. Amaechi, Nkechi L. Chima-Ogbuiyi, Rechner N. Afuh, Divine B. Arrey Agbor, Mohamed Abdirahman Abdi, Nwachukwu O. Nwachukwu, Oluwatobi O. Oderinde, Tochi C. Elendu, Ijeoma D. Elendu, Akinbayo A. Akintunde, Samuel O. Onyekweli, Gloria O. Omoruyi

**Affiliations:** a University of California, Santa Cruz, CA; b Igbinedion University, Okada, Nigeria; c Madonna University, Elele, Nigeria; d Herzing University, Madison, WI; e University Hospitals, Plymouth, England; f Richmond University Medical Center, Staten Island, NY; g Vinnytsia National Pirogov Medical University, Vinnytsia, Ukraine; h Saint Nicholas Hospital, Surulere, Nigeria; i Obafemi Awolowo University, Ile-Ife, Nigeria; j Imo State University, Owerri, Nigeria; k Babcock University, Ilishan-Remo, Nigeria.

**Keywords:** critical care, diagnostic criteria, guidelines, hyperthyroidism, management, scoring systems, thyroid storm

## Abstract

A thyroid storm is a life-threatening endocrine emergency characterized by severe hyperthyroidism and many systemic manifestations. Prompt recognition and treatment are essential for patient survival. This study evaluates the utility of existing diagnostic criteria and scoring systems for thyroid storm. A comprehensive literature review encompassed articles published up to December 2023. Various diagnostic criteria and scoring systems, such as the Burch–Wartofsky Point Scale and the Japanese Thyroid Association criteria, were critically assessed based on their sensitivity, specificity, and clinical applicability. Our findings reveal that existing diagnostic criteria and scoring systems, although valuable tools, exhibit limitations. They may lack sensitivity in identifying milder cases of thyroid storm or fail to differentiate it from other critical conditions. Furthermore, some criteria rely heavily on subjective clinical Judgment, which can vary among healthcare providers. Future research should focus on refining existing criteria and developing more objective and universally applicable diagnostic tools to address these limitations. Incorporating advanced laboratory markers and modern imaging techniques may enhance diagnostic accuracy. Additionally, a standardized scoring system approach could improve clinical practice consistency. In conclusion, while current diagnostic criteria and scoring systems provide a foundation for identifying thyroid storm, their utility has shortcomings. Advancements in diagnostic methods and a collaborative effort to establish standardized criteria are imperative to enhance the accuracy and reliability of thyroid storm diagnosis, ultimately improving patient outcomes.

## 1. Introduction and background

A thyroid storm is a menacing and life-threatening culmination of hyperthyroidism, where the body’s thyroid gland overproduces thyroid hormones to an extreme degree.^[1]^ This condition, while rare, represents a critical medical emergency that demands immediate recognition and intervention.^[1]^ A thyroid storm is characterized by severe symptoms that affect multiple organ systems, including the heart, nervous system, and gastrointestinal tract. Without swift and appropriate treatment, it can lead to catastrophic consequences, including cardiac arrhythmias, organ failure, and even death.^[1,2]^

Given the gravity of the thyroid storm, the accurate and timely diagnosis of this condition becomes paramount. However, this is a complex task.^[2]^ Various diagnostic criteria and scoring systems have been proposed over the years to assist healthcare providers in identifying thyroid storm cases. These tools aim to consolidate clinical features, laboratory data, and physical findings into a comprehensive assessment that guides treatment decisions. Nevertheless, the utility and effectiveness of these diagnostic criteria and scoring systems have come under scrutiny.^[3]^

This paper delves into the intricate landscape of thyroid storm diagnosis, critically evaluating the existing diagnostic criteria and scoring systems in the context of a narrative review.^[1,2]^ By meticulously examining their strengths and limitations, we seek to shed light on the challenges healthcare professionals face when confronted with this formidable endocrine emergency. In doing so, we pave the way for a deeper understanding of thyroid storm and advocate for improvements in its diagnostic methodologies, ultimately enhancing the prospects for timely and life-saving interventions.^[4]^

### 1.1. Importance of accurate diagnosis in thyroid storm management

Accurate diagnosis in thyroid storm management is a critical imperative with far-reaching implications. Operating within the realm of a life-threatening medical emergency, a thyroid storm involves an excessive release of thyroid hormones into the bloodstream, precipitating rapid and severe health deterioration with potentially fatal consequences. In this high-stakes scenario, accurate diagnosis is the inaugural step toward promptly initiating life-saving interventions.^[1]^

The intricacies of thyroid storm diagnosis are compounded by symptom overlap with various medical conditions, including sepsis, heart failure, and drug intoxication. This intricate web of symptoms poses a diagnostic challenge, where misdiagnosis can result in inappropriate treatments and delays in addressing the underlying thyroid dysfunction or the root cause of the symptoms.^[2]^

Accurate diagnosis unveils its significance further in the realm of tailored treatment. The ability to differentiate between thyroid storm and other hyperthyroid states, such as uncomplicated hyperthyroidism, is paramount. The nuances in the treatment approach demand precision, with tailored interventions addressing the specific nature of the underlying thyroid dysfunction.^[3]^

The specter of complications looms large in thyroid storm, encompassing severe outcomes such as cardiac arrhythmias, organ failure, and central nervous system dysfunction. Prompt and accurate diagnosis emerges as the beacon of hope in minimizing these risks, enabling the timely initiation of targeted therapies like beta-blockers, antithyroid medications, and comprehensive supportive care.^[4]^

Beyond the immediate clinical sphere, accurate diagnosis is pivotal in resource optimization. The demands of thyroid storm management often extend to intensive care units and specialized medications. Accurate diagnosis ensures the reasonable allocation of these resources, preventing unnecessary utilization or delayed distribution to patients genuinely in need.^[5]^

Ultimately, the impact reverberates through patient outcomes. Timely and appropriate treatment, anchored in an accurate diagnosis, becomes the catalyst for stabilizing the patient’s condition, mitigating symptom severity, and substantially enhancing the prospects of a full recovery.^[5]^ In the intricate tapestry of thyroid storm management, accurate diagnosis is not just a procedural step but a linchpin shaping the care trajectory and influencing outcomes.

### 1.2. Overview of the diagnostic criteria and scoring systems used in thyroid storm evaluation

Diagnostic criteria and scoring systems play a pivotal role in evaluating thyroid storm, serving as indispensable tools for healthcare providers to identify and classify this life-threatening condition. Various criteria and scoring systems have been developed to facilitate this crucial diagnostic process. Here is an overview of some commonly used approaches:

The Burch-Wartofsky Point Scale is a widely recognized scoring system that assigns points based on clinical signs and symptoms, encompassing temperature, central nervous system manifestations, cardiovascular symptoms, gastrointestinal symptoms, and precipitating factors. A total score exceeding a certain threshold indicates the presence of a thyroid storm.^[6]^

Japanese Thyroid Association Criteria integrate clinical and laboratory parameters, considering factors such as body temperature, pulse rate, central nervous system dysfunction, gastrointestinal symptoms, and thyroid hormone levels. Meeting specific criteria confirms the diagnosis of thyroid storm.^[7]^

Akamizu Criteria, developed by Japanese researchers, focus on clinical features and thyroid hormone levels. They take into account symptoms like fever, tachycardia, congestive heart failure, and abnormal liver function tests in conjunction with thyroid hormone levels.^[4]^

The Adapted Burch-Wartofsky Criteria is a modification tailored to pediatric patients, considering age-specific vital sign parameters and clinical symptoms to diagnose thyroid storm in children.^[4]^

Laboratory-based criteria heavily rely on thyroid hormone levels, particularly extreme elevations exceeding several times the upper reference range, to confirm thyroid storm.^[5]^

Clinical Judgment becomes paramount in some instances where experienced healthcare providers rely on the overall clinical presentation and severity of symptoms to diagnose thyroid storm, especially when specific criteria or scoring systems may not be directly applicable.^[8]^

Inflammatory Markers, such as C-reactive protein (CRP) and interleukin-6 (IL-6), have emerged in recent research as potential contributors to the diagnostic landscape, as their elevation can be indicative of thyroid storm.^[8]^

### 1.3. Objectives of this study

1. To assess the sensitivity and specificity of existing diagnostic criteria and scoring systems for thyroid storm in identifying actual cases of this life-threatening condition.
2. To evaluate the clinical applicability of various diagnostic tools by assessing their ease of use and reliability in diverse healthcare settings.
3. To identify the limitations and challenges associated with the current diagnostic criteria, including their potential for misdiagnosis or delayed diagnosis.
4. To explore the potential for incorporating advanced laboratory markers and modern imaging techniques into the diagnostic process to enhance accuracy.
5. To examine the inter-rater reliability among healthcare providers in applying existing diagnostic criteria and scoring systems.
6. To analyze the impact of accurate thyroid storm diagnosis on patient outcomes, including mortality rates and preventing unnecessary interventions in patients with similar presentations.
7. To provide recommendations for refining existing diagnostic criteria and scoring systems and developing more objective and universally applicable tools for thyroid storm diagnosis.
8. To promote a standardized approach to thyroid storm diagnosis, enhancing consistency in clinical practice and facilitating more timely and effective management of this critical condition.

## 2. Methodology

### 2.1. Search strategy

We conducted a comprehensive literature search using electronic databases, including PubMed, MEDLINE, and Google Scholar. The search encompassed articles published from January 1, 2010, to December 31, 2023. Keywords such as “thyroid storm,” “diagnostic criteria,” and “scoring systems” were employed to identify relevant studies.

### 2.2. Inclusion and exclusion criteria

Studies included in this review provided insights into diagnostic criteria and scoring systems for thyroid storm. We focused on articles published in peer-reviewed journals, emphasizing relevance to clinical practice. Non-English articles and those lacking full-text availability were excluded.

### 2.3. Data extraction

Relevant data were extracted from selected articles, including diagnostic criteria, scoring systems, and their respective evaluations. Emphasis was placed on synthesizing information regarding the strengths and limitations of each diagnostic approach.

### 2.4. Quality assessment

The quality of selected studies was assessed based on the credibility of the data sources, methodological rigor, and relevance to the topic. Studies with a higher level of evidence, such as systematic reviews, meta-analyses, and large cohort studies, were given precedence in the analysis.

### 2.5. Data synthesis

The extracted data were synthesized to provide a cohesive narrative that critically evaluates existing thyroid storm diagnostic criteria and scoring systems. Comparative analyses highlighted different approaches’ similarities, differences, strengths, and limitations.

### 2.6. Review framework

This narrative review adheres to the Preferred Reporting Items for Systematic Reviews and Meta-Analyses (PRISMA) guidelines, ensuring transparency and methodological rigor in selecting and synthesizing relevant literature.

## 3. Discussion

### 3.1. Historical perspectives on diagnostic criteria for thyroid storm

The historical journey of diagnostic criteria for thyroid storm is a captivating narrative, evolving in sync with our deepening understanding of this life-threatening condition and the march of medical progress. In the late 19th century, the seeds of recognition were planted as physicians discerned a constellation of symptoms—rapid pulse, soaring fever, and mental agitation—linked to severe hyperthyroidism. However, these early diagnostic criteria were rudimentary, grounded in keen clinical observation.^[2]^

Fast forward to the 20th century, a pivotal era where diagnostic criteria assumed a more structured form. Visionary clinicians like Robert B. A. Wartofsky and Basil O. Pruitt Jr. played instrumental roles in refining these criteria during the 1960s and 1970s. This period witnessed the birth of scoring systems like the Burch-Wartofsky Point Scale, introducing a numerical framework to standardize thyroid storm diagnoses.^[3]^

Enter the Japanese Thyroid Association, making significant contributions in the late 20th century with the development of diagnostic criteria that incorporated both clinical and laboratory parameters. These criteria found widespread use, particularly in Japan.^[7]^

Yet, the journey is not without its share of controversies. Disputes have simmered over the sensitivity and specificity of criteria. Thyroid storm, a rare and variably presenting condition, posed challenges in crafting universally applicable criteria. In response, some clinicians navigated the diagnostic landscape relying on their Judgment, acknowledging the inherent limitations of existing criteria.^[4]^

As we step into the modern era, research strides forward. Exploring additional laboratory markers, such as CRP and IL-6, introduces a promising layer to diagnostic accuracy. These markers, reflective of the underlying inflammatory processes in thyroid storm, represent a contemporary leap in our quest for precision.^[4]^ The historical odyssey of thyroid storm diagnostic criteria, marked by innovation and adaptation, now stands at the cusp of further breakthroughs in our ongoing pursuit of effective and nuanced diagnosis.

### 3.2. Commonly used diagnostic criteria for thyroid storm

The Burch-Wartofsky Point Scale is a widely embraced diagnostic instrument for thyroid storm, strategically assigning points based on clinical manifestations to gauge the probability of a thyroid storm. Fever above 100.4 °F (38 °C) accrues the maximum score, emphasizing its role as a significant temperature indicator.^[1]^ The scale delves into Central Nervous System (CNS) manifestations, encompassing agitation, delirium, psychosis, and seizures, with points assigned commensurate with severity.^[1–3]^ Gastrointestinal symptoms such as vomiting, diarrhea, and abdominal pain contribute to the assessment, where points align with symptom intensity. The heart rate, a pivotal sign, is scrutinized for tachycardia, escalating points as the heart rate surpasses defined thresholds.^[1]^ The presence of congestive heart failure and atrial fibrillation also factors into the scoring. Points are allocated for identified precipitating factors, be it infection or noncompliance with antithyroid medications, with a total score above a set threshold (e.g., 45 points) indicative of a thyroid storm diagnosis.^[5]^

On a parallel diagnostic path, the Japanese Thyroid Association criteria, predominantly employed in Japan, merge clinical signs with laboratory parameters for thyroid storm diagnosis.^[7]^ Elevated body temperature, CNS symptoms like agitation and delirium, gastrointestinal manifestations including vomiting and diarrhea, and tachycardia are pivotal criteria. Additionally, abnormalities in thyroid function tests, specifically elevated free thyroxine (FT4) levels, contribute to the diagnostic confirmation.^[7]^ These criteria collectively provide a nuanced and comprehensive approach to identifying thyroid storm, reflecting the evolution of diagnostic methodologies.^[1–7]^

### 3.3. Controversies and variations in diagnostic criteria for thyroid storm

Diagnostic criteria for thyroid storm are marred by controversies and variations, creating a landscape of nuanced challenges:

Temperature thresholds, a cornerstone in many criteria, diverge in defining hyperthermia. For instance, the Burch-Wartofsky Point Scale identifies fever above 100.4 °F (38 °C), while others may deploy slightly different values, fostering disparities in thyroid storm diagnoses.^[5]^

Subjectivity looms over symptoms like CNS manifestations and gastrointestinal symptoms, introducing variability between patients and healthcare providers. The interpretation of symptom severity becomes a subjective realm, influencing scoring and diagnostic outcomes.^[8]^

Laboratory parameters, pivotal in most criteria, introduce variations in defining abnormal thyroid hormone levels. The specific thresholds for abnormality, influenced by diverse laboratory reference ranges and measurement units, contribute to diagnosis discrepancies.^[8]^

Clinical Judgment, often pivotal, adds another layer of variability. Some healthcare providers lean on clinical presentation and Judgment rather than strict adherence to criteria, particularly in cases with atypical or overlapping symptoms, introducing diagnosis variability.^[4]^

Geographical differences further complicate matters. The prevalence of the Japanese Thyroid Association criteria in Japan, differing from criteria in other regions, underlines how regional variations impact the identification and management of thyroid storm.^[7]^

The evolution of knowledge perpetuates variations. Advances in medical understanding prompt updates and revisions of diagnostic criteria, resulting in differences between criteria used at different points in time.^[6]^

The introduction of additional markers, like inflammatory indicators CRP and IL-6, as diagnostic tools, contributes to the complexity. Their inclusion or exclusion varies among criteria and guidelines, shaping a dynamic diagnostic landscape.^[2]^ In this intricate interplay of factors, controversies and variations weave a complex tapestry, underscoring the ongoing challenge of achieving standardized and universally applicable diagnostic criteria for thyroid storm.

### 3.4. Scoring systems for thyroid storm

Scoring systems are essential in assessing thyroid storm severity, providing valuable quantification for healthcare providers dealing with this life-threatening condition. Among these systems, the Burch-Wartofsky Point Scale is one of the most widely embraced, assigning numerical points based on clinical signs and symptoms.^[1]^ Parameters include body temperature, central nervous system manifestations, heart rate, gastrointestinal symptoms, congestive heart failure, atrial fibrillation, and precipitating factors, each allocated specific points according to severity. The cumulative score, calculated from these parameters, exceeding a predefined threshold (e.g., 45 points), suggests a thyroid storm diagnosis.^[1]^

The Japanese Thyroid Association Criteria, developed by the Japanese Thyroid Association, merge clinical and laboratory parameters for thyroid storm diagnosis.^[7]^ Parameters encompass body temperature, central nervous system symptoms, gastrointestinal symptoms, heart rate, and thyroid function test results.^[7]^ Diagnosis hinges on meeting specific criteria for each parameter.

Named after Dr Takashi Akamizu, the Akamizu Criteria focus on clinical features and thyroid hormone levels.^[4]^ Clinical features, including fever, tachycardia, congestive heart failure, and abnormal liver function tests, are considered. Diagnosis involves abnormalities in thyroid hormone levels, particularly elevated FT4.^[4]^

Pediatric-specific scoring systems adapt criteria and parameters for thyroid storm assessment in children. Tailored to the pediatric population, these systems account for age-specific vital sign parameters and clinical symptoms.^[4]^

Some approaches lean heavily on laboratory markers, especially thyroid hormone levels, to confirm thyroid storm diagnosis.^[5]^ Extreme elevations in serum thyroid hormone concentrations, often exceeding several times the upper reference range, may serve as diagnostic criteria.^[5]^

Clinical Judgment, wielded by experienced healthcare providers, becomes crucial when specific criteria or scoring systems may not apply or in atypical cases.^[4]^ It offers a nuanced approach grounded in overall clinical presentation, contributing to the comprehensive landscape of thyroid storm assessment.^[4,9]^

### 3.5. The components of these scoring systems and their significance

The components of thyroid storm scoring systems hold pivotal importance in gauging the severity of this life-threatening condition. In commonly used scoring systems, each component is assigned a specific value or score, and the cumulative score indicates the likelihood of a thyroid storm.^[10,11]^

For the Burch-Wartofsky Point Scale, temperature serves as a crucial parameter. Elevated body temperature, a hallmark of thyroid storm, is assessed by assigning points based on temperature, with higher scores correlating to more elevated temperatures. CNS manifestations, such as agitation and delirium, contribute to the overall clinical picture, with assigned points reflecting the severity of these symptoms. Gastrointestinal symptoms, including vomiting and diarrhea, are acknowledged through assigned points, recognizing their role in assessing severity. Heart rate, particularly tachycardia, a critical feature of thyroid storm, is evaluated by assigning points based on the heart rate. Congestive heart failure and atrial fibrillation, potential complications, are considered in the scoring, as are precipitating factors such as infection or noncompliance with antithyroid medications.

The Japanese Thyroid Association Criteria combine clinical and laboratory parameters. A high fever, a critical feature of a thyroid storm, is a crucial diagnostic criterion. CNS symptoms, like agitation and delirium, are assessed for presence and severity. Gastrointestinal symptoms, such as vomiting and diarrhea, are considered, alongside tachycardia, hallmarks of thyroid storm. Abnormal thyroid hormone levels, particularly elevated FT4, are diagnostic criteria.

The Akamizu Criteria focus on clinical features, including fever, tachycardia, congestive heart failure, and abnormal liver function tests indicative of thyroid storm severity. Abnormalities in thyroid hormone levels, especially elevated FT4, are considered in the diagnosis.

These components, encompassing clinical manifestations and laboratory parameters, provide a comprehensive framework for healthcare providers to evaluate and diagnose thyroid storm severity. Table 1 summarizes the components and significance of commonly used thyroid storm scoring systems, including the Burch-Wartofsky Point Scale, Japanese Thyroid Association Criteria, and Akamizu Criteria.^[1,2,4]^

**Table 1:** Different scoring systems.

Scoring system	Burch-Wartofsky Point Scale	Japanese Thyroid Association Criteria	Akamizu Criteria
Components	- Temperature - CNS manifestations - Gastrointestinal symptoms - Heart rate - Congestive heart failure - Atrial fibrillation - Precipitating factors	- Body temperature - CNS symptoms - Gastrointestinal symptoms - Heart rate - Thyroid function tests	- Clinical features - Thyroid hormone levels
Strengths	- Provides a structured and numerical assessment. - Widely recognized and used. - Incorporates a range of clinical signs and precipitating factors.^[11]^	- Combines clinical and laboratory parameters. - Widely used, especially in Japan. - Incorporates thyroid function tests.	- Focuses on clinical features and thyroid hormone levels. - Developed by a notable thyroid specialist.
Limitations	- Subjectivity in scoring some symptoms. - May only capture some atypical cases. - Scoring cutoffs may vary between institutions.^[11]^	- Regional variation in use. - Subjectivity in scoring CNS symptoms. - Limited to specific laboratory markers.	- May not include all clinical features. - Not as widely recognized globally. - Limited emphasis on laboratory markers.

These scoring systems provide valuable tools for assessing thyroid storm severity. However, their strengths and limitations should be considered, and healthcare providers should use clinical judgment alongside these systems to ensure accurate diagnosis and appropriate treatment. The choice of which system to use may also depend on regional practices and institutional guidelines.^[11–13]^

### 3.6. Utility and clinical application

Diagnostic criteria and scoring systems for thyroid storm serve as practical and effective tools in clinical settings, aiding healthcare providers in assessing the severity of this life-threatening condition.^[6,14]^ These systems offer a standardized approach, allowing for quick identification and categorization of cases. By providing an objective assessment method, they reduce reliance on subjective clinical Judgment alone, facilitating communication among healthcare providers.

Regarding effectiveness, scoring systems like the Burch-Wartofsky Point Scale and the Japanese Thyroid Association Criteria emphasize crucial clinical and laboratory parameters. These systems facilitate early diagnosis, which is essential for timely treatment initiation. They also assist in risk stratification, enabling healthcare providers to decide the required level of care and treatment intensity. Moreover, diagnostic criteria and scoring systems often accompany treatment recommendations, guiding healthcare providers in selecting appropriate interventions.^[1]^

Despite their utility, challenges, and considerations exist. These systems may involve some degree of subjectivity, particularly in assessing symptoms like agitation or gastrointestinal distress. Clinical variation in the presentation of thyroid storm, regional differences in adopting scoring systems, and variations in laboratory interpretation can introduce complexities. Additionally, these scoring systems may only partially capture complex cases with comorbidities.

In clinical settings, healthcare providers typically use diagnostic criteria and scoring systems as initial tools to assess and categorize suspected thyroid storm cases. However, the best practice involves combining these tools with clinical Judgment, considering the broader clinical picture, individual patient factors, and regional practices. This comprehensive approach ensures an accurate evaluation, allowing effective, appropriate, and timely interventions to manage this critical medical condition.^[6]^ Table 2 outlines the guidelines for diagnosing and treating thyroid storm.

**Table 2:** Guidelines for the diagnosis and management of thyroid storm.

Guidelines source	Publication date	Key recommendations	Alignment with diagnostic criteria/scoring systems	Practical implementation in clinical practice
ATA	March 12, 2021	- Provide diagnostic criteria for thyroid storm. - Offer recommendations for the treatment of thyroid storm.^[1]^	Aligns with the Burch-Wartofsky Point Scale and other scoring systems.^[1]^	Helpful in establishing standard diagnostic and treatment protocols according to the ATA’s recommendations.^[1]^
Endocrine Society	June 23, 2022	- Offer guidance on the management of thyroid disorders, including thyroid storm.^[3]^	It may include criteria consistent with established scoring systems.^[3]^	Provides insights into the broader context of thyroid disorder management.^[4]^
SCCM	September 13, 2023	- Focus on managing critically ill patients, which can be relevant in severe thyroid storm cases.^[2]^	May emphasize critical care aspects over specific diagnostic criteria.^[2]^	Relevant when thyroid storm presents in a critical care setting.^[2]^
Institutional or Regional Guidelines	March 9, 2023	- Specific guidelines followed by the healthcare institution or region where the article is applicable.^[1]^	May vary but should be consistent with national or international guidelines.^[1]^	Provides context for local practices and considerations.^[1]^
Pediatric Guidelines	January 1, 2023	- Focus on thyroid storm in pediatric patients, offering age-specific recommendations.^[6]^	Tailored to pediatric populations.	This is essential when discussing thyroid storms in children.
Emergency Medicine Guidelines	August 30, 2022	- Pertinent for the initial assessment and management of thyroid storm in an emergency department.^[4]^	May emphasize rapid evaluation and stabilization.	This is crucial when addressing thyroid storms in an emergency setting.

The ATA guidelines provide diagnostic criteria and treatment recommendations for thyroid storm. These align with established scoring systems and help develop standard protocols.^[1]^ The Endocrine Society offers guidance on thyroid disorders management, including thyroid storm. These guidelines may include criteria consistent with scoring systems and provide a broader context for thyroid disorder management.^[3]^ The SCCM guidelines focus on critically ill patients, which can be relevant in severe thyroid storm cases, especially in a critical care setting.^[2]^ Institutional or regional guidelines may vary but should align with national or international recommendations, providing context for local practices.^[1]^ Pediatric guidelines tailor recommendations for thyroid storm in children, offering age-specific considerations.^[6]^ Emergency medicine guidelines are pertinent for the initial assessment and management of thyroid storms in an emergency department, emphasizing rapid evaluation and stabilization.^[4]^ It is essential to refer to the most current versions of these guidelines for accurate and up-to-date information when addressing thyroid storm in clinical practice or research.

ATA = American Thyroid Association, PET = Positron Emission Tomography, MRI = Magnetic Resonance Imaging, SCCM = Society of Critical Care Medicine.

## 4. Case studies illustrating how these criteria and scoring systems have been applied

### 4.1. Case study 1: Burch-Wartofsky Point Scale in action

*A 45-year-old female with a known history of Graves’ disease presented to the emergency department with severe agitation, confusion, fever, and palpitations. Upon examination, she exhibited a temperature of 104 °F (40 °C), a heart rate of 150 bpm, and clear CNS manifestations, including confusion and agitation. Applying the Burch-Wartofsky Point Scale yielded a total score of 65 points, surpassing the threshold of 45 points. This high score strongly suggested thyroid storm, prompting immediate intervention and emphasizing the practical application of scoring systems in confirming the severity of the condition.*

### 4.2. Case study 2: Japanese Thyroid Association Criteria in action

*In another scenario, a 32-year-old male with untreated hyperthyroidism due to Graves’ disease presented to the emergency department with vomiting, diarrhea, and palpitations. His temperature measured 102 °F (38.9 °C), his heart rate was 140 bpm, and he exhibited gastrointestinal symptoms and signs of thyroid toxicity. Applying the Japanese Thyroid Association Criteria, the patient met the criteria for thyroid storm due to elevated body temperature, gastrointestinal symptoms, and tachycardia. Further thyroid function tests were pending but clinically suggestive.* This case illustrates the relevance of utilizing different diagnostic criteria and scoring systems to assess and categorize patients suspected of having thyroid storm. It emphasizes the role of scoring systems in guiding clinical decision-making, initiating appropriate treatment, and determining the severity of the condition.

These case studies vividly demonstrate how healthcare providers can apply various diagnostic criteria and scoring systems to assess and categorize patients suspected of having thyroid storm. The practical application of scoring systems, such as the Burch-Wartofsky Point Scale and the Japanese Thyroid Association Criteria, is crucial in guiding clinical decision-making, initiating timely treatment, and gauging the severity of the condition. However, they also highlight the importance of clinical Judgment in interpreting results and tailoring treatment to individual patient needs, showcasing the practical aspects of diagnostic challenges in real-world scenarios.

### 4.3. The impact of using these tools on early diagnosis and timely intervention

Implementing diagnostic criteria and scoring systems for thyroid storm represents a transformative approach in healthcare, revolutionizing the landscape of early diagnosis and intervention.^[3,15]^ These tools serve as a compass, navigating healthcare providers through a standardized assessment that transcends the limitations of subjectivity. Objectivity takes center stage as numerical values dance across the clinical canvas, offering a precise evaluation beyond individual interpretations.

Early recognition, akin to catching the first notes of a crescendo, becomes a hallmark.^[3]^ Scoring systems, emphasizing vital signs like temperature and heart rate, act as vigilant sentinels, sounding the alarm at the earliest whispers of a thyroid storm.^[3]^ Risk stratification emerges as a guiding light, illuminating the path toward tailored interventions for patients teetering on the precipice of complications.^[3]^

Similar to a well-choreographed performance, treatment guidance unfolds seamlessly alongside these tools.^[3]^ Accompanied by treatment guidelines, they ensure that the stage is set for evidence-based therapies, with beta-blockers and antithyroid medications taking the lead.^[3]^ Communication becomes a symphony, harmonizing healthcare providers’ language and documenting the rationale behind diagnosis and treatment decisions, creating a shared narrative within the healthcare team.^[3]^ Yet, the impact extends beyond the spotlight of initial diagnosis. These tools become vigilant guardians, guarding against underdiagnosis through specific cutoff thresholds.^[3]^ They watch the patient’s progress, enabling a dynamic response to evolving situations.^[15]^ In this orchestrated dance of assessment and intervention, these tools transform the narrative of thyroid storm management, ensuring that every patient’s story concludes with a note of recovery and resilience.^[3]^

### 4.4. Challenges and controversies

Navigating the realm of thyroid storm diagnosis comes with a symphony of challenges and complexities echoing throughout the corridors of healthcare. Subjectivity becomes a subtle note in the scoring system composition, where assessing symptoms like central nervous or gastrointestinal manifestations introduces a variable melody between healthcare providers.^[3]^ This variation in scoring adds nuances to the diagnostic process, potentially leading to discrepancies in thyroid storm identification.

The variability in clinical presentation becomes a dynamic interplay, a challenge that echoes across the stage of thyroid storm diagnosis.^[3]^ The condition’s diverse symptoms and severity levels create a narrative that is challenging to capture entirely using standardized criteria. In this complexity, atypical or milder cases may remain elusive due to the rigid structures of diagnostic tools, leading to potential delays in diagnosis and intervention.^[3]^

Like regional accents in a theatrical performance, regional differences add another layer to the thyroid storm saga.^[3]^ Different regions and institutions adopting distinct criteria or scoring systems introduce variations in the diagnosis and management of thyroid storm. Healthcare providers donned as actors on this diverse stage must be attuned to and adept at adapting to local practices to ensure optimal patient care.

Laboratory variability contributes its composition to the diagnostic symphony, where the interpretation of thyroid hormone levels waltzes within the confines of reference ranges.^[3]^ Differences in these ranges can introduce inconsistencies in scoring, impacting the diagnostic journey and potentially leading to discordant diagnoses.

A delicate balance is struck in the dance between scoring systems and clinical Judgment.^[3]^ The structured notes of the scoring systems harmonize with the nuanced melody of Clinical Judgment. Yet, challenges arise when this balance tilts, especially in complex cases where overreliance on scoring systems may play a discordant note, potentially resulting in misdiagnosis or delayed intervention.^[3]^

The sensitivity of scoring systems becomes a key element, a note that may falter in capturing all cases of thyroid storm, particularly those with subtle or atypical presentations.^[3]^ Some patients may remain outside the predefined criteria, lingering in the shadows of delayed diagnosis and treatment.

Like intricate choreography, complex cases introduce additional layers to the thyroid storm narrative.^[3]^ Patients with comorbidities or complicating factors may dance to a different rhythm, challenging scoring systems to account for these complexities fully. Here, clinical Judgment must sway gracefully to adapt to the unique needs of each patient.^[3]^

As our understanding of the thyroid storm evolves, a silent evolution echoes through the criteria and scoring systems.^[3]^ The need for updates and revisions becomes a backdrop, ensuring that the diagnostic stage mirrors the latest medical knowledge, avoiding the dissonance of outdated criteria potentially affecting diagnostic accuracy. This evolution becomes essential, bringing the thyroid storm diagnostic symphony in tune with the ever-advancing landscape of medical science.^[3]^

Pediatric patients introduce their challenges as the youngest performers in this diagnostic theater.^[3]^ Some scoring systems, crafted for the adult stage, may only partially resonate in the pediatric realm. A need for pediatric-specific criteria or adaptations emerges, highlighting the importance of tailoring diagnostic approaches to the unique characteristics of pediatric patients.^[3]^

The quest for accurate and timely thyroid storm diagnosis persists in this intricate ballet of challenges and controversies. Healthcare providers, as conductors of this diagnostic orchestra, must navigate these challenges with precision, adapting their approach to each patient’s unique melody.

### 4.5. Situations where the criteria may not apply or may lead to misdiagnosis

In the intricate landscape of thyroid storm diagnosis, there exist scenarios where the established diagnostic criteria and scoring systems face challenges and potential pitfalls. The complexity and variations in clinical presentations contribute to situations where these tools may be less effective or, in some instances, lead to misdiagnosis.^[15,16]^ Thyroid storm’s atypical presentation, particularly in elderly patients or those with underlying medical conditions, poses a formidable challenge. Manifesting with milder symptoms or unusual clinical forms, patients might not meet predefined criteria, potentially resulting in underdiagnosis.

The concept of subclinical thyroid storm introduces another layer of complexity. In cases where patients exhibit biochemical evidence of severe hyperthyroidism without overt clinical symptoms, scoring systems, primarily attuned to clinical manifestations, may overlook these subtler presentations. Euthyroid sick syndrome, associated with severe non-thyroidal illness, mirrors thyroid storm symptoms but lacks significantly elevated thyroid hormone levels. Scoring systems focusing on hormone levels may misclassify these patients, blurring the lines between distinct conditions.

Patients navigating multiple medical conditions, such as sepsis, heart failure, or drug intoxication, create diagnostic dilemmas. The challenge lies in the inability of diagnostic criteria and scoring systems to effectively differentiate between thyroid storm and these concurrent conditions, potentially leading to misdiagnosis.^[17]^ Certain medications, with their capacity to mimic thyroid storm symptoms, introduce a pharmacological twist. Scoring systems might not adequately account for drug-induced symptoms, resulting in false-positive diagnoses. The realm of pediatric patients, characterized by unique vital sign ranges and clinical presentations, adds another layer of complexity. Scoring systems designed for adults may not seamlessly adapt to the pediatric landscape, raising concerns about their suitability in assessing thyroid storm in younger patients. Elderly patients, with their distinctive clinical nuances, may not conform to classic thyroid storm signs. The challenge arises when scoring systems designed for a younger demographic encounter elderly patients who deviate from the expected clinical profile.^[18]^

Laboratory variability, influenced by variations in reference ranges, casts a shadow over the diagnostic process. Inconsistent results in interpreting thyroid function tests, a cornerstone of scoring systems, may emerge due to these variations. Complex cases where patients grapple with multiple endocrine disorders or uncommon triggers for thyroid storm challenge the existing diagnostic criteria.^[19]^ These cases, refusing to fit into established criteria neatly, pose a puzzle for healthcare providers, making diagnosis more challenging. The evolution of knowledge, a dynamic force shaping our understanding of the thyroid storm, adds a temporal dimension to these challenges. As insights progress, diagnostic criteria may require updates to align with the latest experience, rendering outdated criteria potentially less effective in capturing the intricacies of thyroid storm diagnosis.^[20]^

### 4.6. Present controversies or debates within the medical community regarding their utility

Controversies and debates within the medical community surround the utility of diagnostic criteria and scoring systems for thyroid storm, touching upon several critical points of contention.^[17,18]^ Sensitivity versus specificity is an ongoing debate, with arguments about the potential trade-off. Some contend that stricter criteria with higher specificity might miss milder cases, while more sensitive criteria could lead to overdiagnosis and unnecessary treatment. The balance between clinical judgment and scoring systems is another focal point. Healthcare providers differ in their reliance on either, given the variable presentations of thyroid storm that may need to be more neatly fit into scoring criteria. While some emphasize the objectivity and consistency scoring systems provide, others underscore the importance of clinical Judgment.

The appropriateness of using the same criteria for all age groups and populations is debatable. Pediatric patients, the elderly, and those with coexisting conditions may present differently, challenging the uniform application of standardized criteria. Debates also emerge around scoring systems’ emphasis on thyroid hormone levels. Critics argue that solely relying on these levels may lead to misdiagnosis, particularly in cases of euthyroid sick syndrome or non-thyroidal illness.^[21]^

Regional variation in criteria and scoring systems adoption introduces potential inconsistencies in diagnosis and management. The need for a standardized global approach is a matter of discussion. As our understanding of the thyroid storm evolves, ongoing debate surrounds whether existing criteria adequately reflect the latest medical knowledge. Some argue for frequent updates to incorporate new insights, while others emphasize the importance of stability for clinical practice.^[22]^ The debate extends to the appropriate point for initiating treatment based on scoring systems. While higher scores indicate severe cases requiring immediate intervention, managing patients with borderline scores remains a subject of debate. Recent research exploring the utility of inflammatory markers like CRP and IL-6 in diagnosing thyroid storm sparks debate. Ongoing discussions are integrating these markers into existing criteria and understanding their clinical significance.^[23]^

Controversies also extend to the impact of these criteria and scoring systems on patient outcomes. Some argue that their use leads to earlier diagnosis and improved outcomes, while others question whether they adequately capture all cases of thyroid storm. These ongoing controversies and debates highlight the complex nature of diagnosing and managing thyroid storm. While these tools offer valuable guidance, their limitations and potential for variation underscore the need for ongoing research, collaboration, and a nuanced approach to clinical decision-making in thyroid storm cases.^[24]^

### 4.7. Recent advances and emerging trends

Recent advances and emerging trends in thyroid storm diagnosis reflect a dynamic landscape in medical research and technology. Inflammatory markers, such as CRP and IL-6, have become a focal point, with elevated levels associated with the underlying inflammatory processes in the thyroid storm. Integrating these markers into diagnostic criteria holds promise for enhancing accuracy.^[19]^ Machine learning algorithms and artificial intelligence (AI) are gaining recognition for their potential to aid thyroid storm diagnosis. These technologies analyze diverse patient data, including clinical symptoms, vital signs, and laboratory results, identifying patterns contributing to early diagnosis.^[3]^ Exploration into genetic and molecular biomarkers associated with thyroid storm is underway, offering the potential for more targeted diagnostic tests and therapies.^[6]^ Emerging trends also emphasize patient-specific risk assessment, considering individual factors like age, comorbidities, and specific thyroid disease etiology to predict the likelihood of developing thyroid storm.^[12]^ The COVID-19 pandemic has accelerated the adoption of telemedicine and remote monitoring, with these technologies increasingly used for monitoring patients with thyroid disorders. This allows for early intervention and timely adjustments to treatment plans.^[7]^

Recognizing the need for global collaboration and standardization, efforts are underway to develop consensus guidelines and criteria for diagnosing and managing thyroid storm universally, reducing regional variations.^[4]^ Advanced imaging techniques, including thyroid scintigraphy and ultrasound, are being explored for their role in diagnosing thyroid storm. These modalities provide insight into thyroid gland activity and inflammation.^[5]^

An emerging trend focuses on patient education and awareness, emphasizing the importance of raising awareness among patients and healthcare providers about the signs and symptoms of thyroid storm. Prompt recognition by patients and timely medical attention are crucial elements in effectively managing thyroid storm.^[7]^ As ongoing research and innovation continue, staying updated with the latest developments in thyroid storm diagnosis is vital. New guidelines and emerging technologies can improve diagnostic accuracy, enhance risk assessment, and refine management strategies for this critical medical condition. Healthcare professionals should remain informed to provide the best care for patients at risk of thyroid storm. Table 3 provides a comprehensive overview of recent advances and emerging trends in thyroid storm diagnosis, summarizing key developments in inflammatory markers, machine learning, Next-Generation Sequencing, metabolomics, and advanced imaging.

**Table 3:** Recent advances and emerging trends in thyroid storm diagnosis.

Diagnostic advancement	Key features	Potential impact	Current status	Future implications
Inflammatory markers (CRP, IL-6)	Elevated levels associated with thyroid storm inflammation	Enhanced accuracy in diagnosis	Explored in research, potential integration into criteria	Promising for precision
Machine learning and AI	Analyzing diverse patient data for early diagnosis	Improved early diagnosis, pattern recognition	Gaining recognition, ongoing development	Potential transformative role
NGS	Comprehensive genetic analysis	Identifying genetic variants linked to thyroid disorders	Under exploration for thyroid storm	Personalized risk assessment
Metabolomics	Studying metabolic profiles associated with thyroid storms	Insights into condition and diagnosis	Investigational, potential future application	Understanding metabolic signatures
Advanced imaging (PET, MRI)	Visualizing thyroid gland activity and inflammation	Complementing traditional criteria	Explored for diagnostic use	Valuable insights into thyroid storm pathophysiology

The table presents a comprehensive overview of recent advances and emerging trends in thyroid storm diagnosis. Each diagnostic advancement is outlined with crucial features, potential impact, current status, and future implications. From exploring of inflammatory markers like CRP and IL-6 to the promising role of machine learning and AI in early diagnosis, the table encapsulates the evolving landscape of diagnostic methodologies. Whether it is the potential precision offered by NGS or the valuable insights from studying metabolic profiles through Metabolomics, each advancement brings unique contributions to refining thyroid storm diagnosis. Including advanced imaging techniques such as PET and MRI further highlights their complementary role in traditional diagnostic criteria. This comprehensive overview serves as a guide to understanding the ongoing transformation in thyroid storm diagnosis, providing insights into the potential future directions of these diagnostic tools.

AI = artificial intelligence; CRP = C-reactive protein, IL-6 = interleukin-6; NGS = next-generation sequencing.

### 4.8. New diagnostic tools, technologies, or research that may impact the evaluation of thyroid storm

Ongoing research unlocks new frontiers in evaluating thyroid storm, with several innovative tools and technologies poised to reshape diagnostic approaches. Biomarker discovery is a focal point, aiming to identify specific markers associated with thyroid storm, ranging from thyroid hormone levels and inflammatory markers to genetic and molecular signatures. Such discoveries could pave the way for more precise diagnostic tests.^[15]^ Next-generation sequencing technologies comprehensively analyze a patient’s genetic makeup. Researchers explore next-generation sequencing to identify genetic variants linked to thyroid disorders, potentially enhancing personalized risk assessment and diagnosis.^[16]^

Metabolomics, which studies small molecules in biological fluids, is being leveraged to investigate metabolic profiles associated with thyroid storm. Identifying unique metabolic signatures holds promise for gaining insights into the condition and its diagnosis.^[5]^ Advanced imaging modalities like positron emission tomography and magnetic resonance imaging are explored for visualizing thyroid gland activity and inflammation, complementing traditional diagnostic criteria.^[4]^

AI and machine learning algorithms are developing to analyze complex datasets, including clinical symptoms, laboratory results, and imaging findings. Their role is crucial in aiding early diagnosis and predicting the risk of thyroid storm.^[5]^ Point-of-care testing devices for thyroid hormones and relevant biomarkers are developing, offering rapid diagnostic capabilities in emergency settings where timely intervention is paramount.^[18]^ Patient monitoring apps and wearable devices enable remote tracking of vital signs and symptoms. These technologies can potentially monitor individuals at risk for thyroid storm, facilitating early intervention.^[4]^ Immunological markers and autoantibodies are under investigation for their role in thyroid storm. These markers may assist in identifying at-risk individuals, contributing to a more comprehensive diagnostic approach.^[7]^

International collaborations among medical societies and researchers aim to develop standardized diagnostic criteria and guidelines for thyroid storm. This global effort seeks to reduce regional variations in diagnosis and management.^[6]^ Patient education and empowerment programs are underway to educate individuals about thyroid disorders, symptoms, and the importance of seeking timely medical attention. Empowering patients to recognize warning signs is critical for thyroid storm prevention.^[3]^ As research in these areas progresses, the potential transformation of thyroid storm evaluation becomes evident, promising more accurate, personalized, and timely diagnostic methods. Staying informed about these emerging tools and technologies enables healthcare professionals to diagnose and manage thyroid storm effectively.^[25]^

### 4.9. Recommendations for healthcare professionals

Healthcare professionals are advised to broaden their knowledge by familiarizing themselves with various thyroid storm diagnostic criteria and scoring systems, recognizing that different institutions or regions may employ diverse approaches. While scoring systems offer valuable insights, clinical Judgment remains pivotal, requiring consideration of the overall clinical context, patient history, and potential atypical presentations. Staying abreast of the latest guidelines and research in thyroid storm diagnosis is crucial, necessitating regular participation in workshops, conferences, and educational programs to enhance diagnostic skills continually.

Interdisciplinary collaboration is critical in thyroid storm cases, often involving endocrinology, cardiology, and critical care specialists. Collaborating with multiple specialties ensures a comprehensive evaluation and management approach. Healthcare professionals should adopt an individualized assessment strategy, acknowledging that patient characteristics such as age and comorbidities can influence thyroid storm presentations. This tailored approach ensures that assessment and management align with individual patient needs. Remaining open to evolving knowledge is essential, considering that understanding the thyroid storm continuously advances. Healthcare professionals are encouraged to integrate new diagnostic tools, biomarkers, and research findings into their practice. Patient education plays a crucial role, particularly in informing individuals with known thyroid disorders about the signs and symptoms of thyroid storm, emphasizing the importance of seeking prompt medical attention when warning signs arise.

Effective communication within the healthcare team is vital. Healthcare professionals should ensure that all team members are well-versed in diagnostic criteria and understand the rationale behind diagnosis and treatment decisions. This collaborative and informed approach contributes to comprehensive and well-coordinated care for patients at risk of or experiencing thyroid storm.

### 4.10. Areas for future research and improvement

Future research efforts should focus on specific biomarker discovery associated with thyroid storm, aiming to develop more precise and early diagnostic tests. Global standardization of diagnostic criteria and scoring systems is crucial to reducing regional variations and ensuring consistency in thyroid storm diagnosis and management worldwide. Tailored research for pediatric and elderly populations is essential, aiming to create age-specific diagnostic criteria for normal variations in vital signs and clinical presentation. Investigating the effectiveness of remote monitoring and telemedicine technologies in early thyroid storm detection, particularly in patients with preexisting thyroid disorders, is recommended. The role of advanced imaging techniques, such as positron emission tomography and magnetic resonance imaging, in thyroid storm diagnosis and risk assessment should be explored further. The ongoing development of AI algorithms to analyze complex patient data for early detection and prediction of thyroid storm is a promising avenue.

Research on integrating patient-reported symptoms and experiences into diagnostic criteria could provide a more comprehensive assessment of thyroid storm. Genomic and molecular studies are essential to understanding the underlying mechanisms and identifying potential targets for diagnosis and treatment. Public awareness campaigns are needed to educate individuals about thyroid disorders and encourage timely medical attention. Exploring strategies for preventing thyroid storm in high-risk populations, including improving adherence to antithyroid medications and optimizing underlying thyroid conditions, is critical for future research. Addressing these areas will enhance healthcare professionals’ ability to accurately and promptly diagnose thyroid storm, improve patient outcomes, and reduce morbidity and mortality associated with this critical medical condition.

## 5. Conclusion

This article delves into the critical realm of thyroid storm diagnosis, underscoring its life-threatening nature and the importance of precise assessment and timely intervention. Key highlights encompass the severe and urgent manifestation of hyperthyroidism that the thyroid storm represents, demanding immediate medical attention.

Emphasis has been placed on the pivotal role of accurate and timely diagnosis ineffective management, acknowledging the potential for severe complications and mortality if the diagnosis is missed or delayed. Various diagnostic criteria and scoring systems, such as the Burch-Wartofsky Point Scale, Japanese Thyroid Association Criteria, and Akamizu Criteria, have been explored as structured approaches to assess the severity of thyroid storm, promoting objective evaluation and risk stratification. Navigating the challenges and controversies surrounding thyroid storm diagnosis, including subjectivity in scoring, variations in clinical presentation, and regional differences in criteria, has been elucidated. Controversies around the balance between sensitivity and specificity and the role of clinical Judgment versus scoring systems have also been discussed. Recent advances in thyroid storm diagnosis, encompassing the utilization of inflammatory markers, artificial intelligence, genetic research, and personalized risk assessment, have been highlighted to enhance accuracy and early detection. Recommendations for healthcare professionals to familiarize themselves with multiple diagnostic systems, exercise clinical Judgment, stay updated through education and training, and collaborate across specialties for comprehensive patient care have been emphasized.

Future directions in thyroid storm research and improvement include biomarker discovery, global standardization of criteria, age-specific diagnostic criteria, exploration of remote monitoring technologies, integration of artificial intelligence, consideration of patient-reported outcomes, and implementation of public awareness campaigns. These areas promise to enhance accuracy in diagnosis, improve patient outcomes, and mitigate the morbidity and mortality associated with this critical medical condition.

*Conclusion and call to action*: Accurate diagnosis is the linchpin of effective thyroid storm management. The evolving landscape of thyroid storm diagnosis, marked by emerging technologies and a growing understanding of its complexities, underscores the need for healthcare professionals to adapt and stay informed. As we navigate the challenges and controversies in this field, we must prioritize the early identification and timely intervention of thyroid storm to protect the lives and well-being of patients facing this life-threatening condition. In an ever-evolving medical landscape, the call to action remains clear: continuous education, collaboration, and research are the keys to improving the diagnosis and management of thyroid storm, ultimately saving lives and enhancing patient outcomes.

## Acknowledgments

We sincerely thank the medical community, researchers, and healthcare professionals who have dedicated their efforts to advancing our understanding of thyroid storm and improving diagnostic approaches. Their commitment to research, education, and patient care contributes significantly to the field and benefits those at risk of this life-threatening condition. We also acknowledge the contributions of patients and their families, whose experiences and insights are invaluable in shaping our understanding of thyroid disorders. Their resilience and willingness to participate in research are crucial for advancing medical knowledge and improving outcomes. Furthermore, we appreciate the collaborative efforts of institutions, organizations, and societies that foster global collaboration, standardization, and innovation in thyroid storm diagnosis and management. These collective endeavors contribute to the progress and evolution of diagnostic criteria, scoring systems, and treatment strategies. Our Acknowledgments extends to the educators and mentors who guide healthcare professionals in their continuous learning journey. The dissemination of knowledge through workshops, conferences, and educational programs plays a vital role in enhancing diagnostic skills and promoting effective patient care. Lastly, we express our appreciation for the tireless work of all healthcare professionals who, through their dedication and expertise, strive to ensure accurate and timely diagnoses, ultimately improving the lives of individuals at risk of thyroid storm.

## Author contributions

Conceptualization, data curation, formal analysis, funding acquisition, investigation, methodology, project administration: Chukwuka Elendu, Dependable C. Amaechi, Emmanuel C. Amaechi, Tochi C. Elendu, Ijeoma D. Elendu.

Resources, software, supervision, validation, visualization, writing – original draft, writing – review & editing: Chukwuka Elendu, Dependable C. Amaechi, Emmanuel C. Amaechi, Nkechi L. Chima-Ogbuiyi, Rechner N. Afuh, Divine B. Arrey Agbor, Mohamed Abdirahman Abdi, Nwachukwu O. Nwachukwu, Oluwatobi O. Oderinde, Tochi C. Elendu, Ijeoma D. Elendu, Akinbayo A. Akintunde, Samuel O. Onyekweli, Gloria O. Omoruyi.

## References

[1] Pokhrel B Aiman W Bhusal K. Thyroid storm. [Updated 2022 October 6]. In: StatPearls. Treasure Island, FL: StatPearls Publishing. 2023. 28846289

[2] Macvanin MT Gluvic ZM Zaric BL. New biomarkers: prospect for diagnosis and monitoring of thyroid disease. Front Endocrinol (Lausanne). 2023;14:1218320. 37547301 10.3389/fendo.2023.1218320 PMC10401601

[3] Islam SS Haque MS Miah MSU. Application of machine learning algorithms to predict the thyroid disease risk: an experimental comparative study. PeerJ Comput Sci. 2022;8:e898. 10.7717/peerj-cs.898 PMC9044232 35494828

[4] Aversano L Bernardi ML Cimitile M. A systematic review on artificial intelligence techniques for detecting thyroid diseases. PeerJ Comput Sci. 2023;9:e1394. 10.7717/peerj-cs.1394 PMC10280452 37346658

[5] Abisad DA Glenn Lecea EM Ballesteros AM. Thyroid storm in pediatrics: a systematic review. J Pediatr Endocrinol Metab. 2022;36:225–33. 36318760 10.1515/jpem-2022-0309

[6] De Almeida R McCalmon S Cabandugama PK. Clinical review and update on the management of thyroid storm. Mo Med. 2022;119:366–71. 36118802 PMC9462913

[7] Bourcier S Coutrot M Kimmoun A. Thyroid storm in the ICU: a retrospective multicenter study. Crit Care Med. 2020;48:83–90. 31714398 10.1097/CCM.0000000000004078

[8] Chiha M Samarasinghe S Kabaker AS. Thyroid storm: an updated review. J Intensive Care Med. 2015;30:131–40. 23920160 10.1177/0885066613498053

[9] Ritter K Wolfe C. Thyroid storm. J Educ Teach Emerg Med. 2020;5:O1–O30. 10.21980/J8RW71 PMC10334447 37465337

[10] Agarwal S Bychkov A Jung CK. Emerging biomarkers in thyroid practice and research. Cancers (Basel). 2021;14:204. 35008368 10.3390/cancers14010204 PMC8744846

[11] Negrini S Donzelli S Negrini A. Feasibility and acceptability of telemedicine to substitute outpatient rehabilitation services in the COVID-19 emergency in Italy: an observational everyday clinical-life study. Arch Phys Med Rehabil. 2020;101:2027–32. 32800748 10.1016/j.apmr.2020.08.001 PMC7422840

[12] Kichloo A Albosta M Dettloff K. Telemedicine, the current COVID-19 pandemic and the future: a narrative review and perspectives moving forward in the USA. Fam Med Community Health. 2020;8:e000530. 32816942 10.1136/fmch-2020-000530 PMC7437610

[13] Angell TE Lechner MG Nguyen CT. Clinical features and hospital outcomes in thyroid storm: a retrospective cohort study. J Clin Endocrinol Metab. 2015;100:451–9. 25343237 10.1210/jc.2014-2850

[14] Ganesh BB Bhattacharya P Gopisetty A. Role of cytokines in the pathogenesis and suppression of thyroid autoimmunity. J Interferon Cytokine Res. 2011;31:721–31. 21823922 10.1089/jir.2011.0049 PMC3189552

[15] Ma Y Li H Liu J. Impending thyroid storm in a pregnant woman with undiagnosed hyperthyroidism: a case report and literature review. Medicine (Baltim). 2018;97:e9606. 10.1097/MD.0000000000009606 PMC5779755 29504986

[16] Di Cerbo A Quagliano N Napolitano A. Comparison between an emerging point-of-care tool for TSH evaluation and a centralized laboratory-based method in a cohort of patients from Southern Italy. Diagnostics (Basel). 2021;11:1590. 34573932 10.3390/diagnostics11091590 PMC8471571

[17] Wang W Shen C Zhao Y. Identification and validation of potential novel biomarkers to predict distant metastasis in differentiated thyroid cancer. Ann Transl Med. 2021;9:1053. 34422965 10.21037/atm-21-383 PMC8339873

[18] Gavin KM Kreitzberg D Gaudreau Y. Identification and management of thyroid dysfunction using at-home sample collection and telehealth services: retrospective analysis of real-world data. J Med Internet Res. 2023;25:e43707. 37252757 10.2196/43707 PMC10265408

[19] Vidouris M Worth C Patel L. Notes for the general paediatrician: managing thyrotoxicosis in children and young people. BMJ Paediatr Open. 2022;6:e001582. 10.1136/bmjpo-2022-001582 PMC9685199 36645751

[20] Reddy G Livingston J Gandhi D. Thyrotoxic crisis in the absence of risk factors: a case report. Cureus. 2023;15:e39972. 37416045 10.7759/cureus.39972 PMC10321309

[21] Bini F Pica A Azzimonti L. Artificial intelligence in thyroid field-a comprehensive review. Cancers (Basel). 2021;13:4740. 34638226 10.3390/cancers13194740 PMC8507551

[22] Li X Chen J Li Z. PICU treatment of 3 cases of pediatric thyroid storm: case series and literature review. Medicine (Baltim). 2023;102:e33447. 10.1097/MD.0000000000033447 PMC10082310 37026965

[23] Almohammed HI Mansour S Alhulwah AH. Scintigraphy has the potential to replace thyroid stimulating hormone and ultrasonography in hyperthyroidism diagnosis. Saudi J Biol Sci. 2020;27:1722–5. 32565688 10.1016/j.sjbs.2020.05.015 PMC7296473

[24] Mahadik K Choudhary P Roy PK. Study of thyroid function in pregnancy, its feto-maternal outcome; a prospective observational study. BMC Pregnancy Childbirth. 2020;20:769. 33302910 10.1186/s12884-020-03448-z PMC7726876

[25] Pegoraro V Bidoli C Dal Mas F. Cardiology in a digital age: opportunities and challenges for e-Health: a literature review. J Clin Med. 2023;12:4278. 37445312 10.3390/jcm12134278 PMC10342385

